# Diversity and sources of floral microbial communities

**DOI:** 10.1128/aem.00323-26

**Published:** 2026-08-03

**Authors:** Muhammad Qaisar Naeem Khan, Wen-Qian Xiang, Ming-Xun Ren

**Affiliations:** 1 International Joint Center for Terrestrial Biodiversity around South China Sea of Hainan Province, School of Ecology, Hainan University 74629, Haikou, China; 2 Ministry of Education Key Laboratory for Genetics and Germplasm Innovation of Tropical Special Forest Trees and Ornamental Plants, Hainan University 74629, Haikou, China; The Pennsylvania State University, University Park, Pennsylvania, USA

**Keywords:** floral microbiome, plant-pollinator interactions, floral biology, microbial transmission

## Abstract

Flowers serve as a key ecological interface where microorganisms, plants, and pollinators interact, yet the processes shaping flower microbiota remain insufficiently synthesized. In this review, we summarize the current literature on the assembly and distribution of floral microbiota across floral organs through three major transmission pathways: insect vectors, bird visitation, and aerial deposition. We assess the organ-specific distribution and transmission pathways of microbial taxa across nectar, stigma, petals, pollen, and anthers, and estimate the relative importance of each of these vectors in microbial dispersal. Our analysis reveals a strong research bias toward nectar and stigma, which collectively dominate the studies of floral microbiota. Based on the currently available literature, insect pollinators, especially the honeybees and bumblebees, emerge as the most frequently reported agents of microbial transfer by repeated deposition of microbes onto floral organs. Bird-mediated transmission, despite its geographic restriction, plays a key role in ornithophilous systems, whereas aerial deposition appears to have a comparatively limited influence. We also emphasize the effect of floral microbes on flower attractiveness, phenotypic changes, and the systemic microbial movement within plants. This review incorporates flower ecology, microbiology, and pollination biology, and points out how flowers act as microbial gateways between the aboveground and belowground parts of plants, and how flowers remain underappreciated for their role in systemic interaction between plants and microbes.

## INTRODUCTION

Flowers are among the most ecologically important structures of plants, serving as the reproductive interface between the angiosperms and the biotic environment. Traditionally, researchers have focused on the visual, olfactory, and structural characteristics of flowers to attract pollinators. However, flowers are increasingly recognized as microbial habitats that harbor taxonomically diverse communities of bacteria, fungi, and yeasts, although microbial diversity within individual flowers is often relatively low because of intense environmental filtering (1, 2). These microbial communities, collectively referred to here as the floral microbiota, inhabit various floral organs, most commonly the petals, anthers, stigmas, and particularly the nectar. This floral habitat, known as the anthosphere, represents a distinct aboveground ecological niche. Despite the short lifespan of flowers, microbes associated with plants in the anthosphere may have significant impacts on plant reproduction, pollinator behavior, and broader ecosystem processes. The floral microbiota is not a random assemblage; rather, it is structured by several selective forces, including floral features, microclimatic influences, and most importantly, patterns of microbial dispersal (2). Microbial isolates associated with flowers include nectar-specialist yeasts, such as *Metschnikowia reukaufii*, as well as floral surface-associated bacteria, including *Acinetobacter*, *Pseudomonas*, and *Fructobacillus*, and yeasts like *Candida* spp. These microbes have specialized traits that allow them to survive in harsh floral environments, which are characterized by high levels of osmotic pressure, low nitrogen availability, and elevated exposure to UV radiation and secondary metabolites (3, 4).

The presence of microbes in flowers is not incidental but functionally significant. Floral microorganisms actively modify plant-pollinator interactions through their metabolic activities. For example, nectar microbes can hydrolyze sucrose into glucose and fructose, lower nectar pH through the production of organic acids, and change the nectar viscosity (5). Volatile organic compounds may also be produced or broken down by microbial metabolism, modifying floral scent profiles and influencing their attractiveness, resulting in pollinator attraction (5). Experimental studies have shown that these changes mediated by microbes may either attract or repel pollinators depending on microbial identity and metabolite concentration, which in turn can affect visitation rates and pollination success (6).

In some cases, floral microbes can also provide protective mutualistic effects by outcompeting pathogenic microbes through competitive exclusion or by producing antimicrobial compounds (7). Conversely, certain microbes may act as pathogens or transmission agents that contribute to the development of floral diseases, including fire blight caused by *Erwinia amylovora* (8).

**Fig 1:**
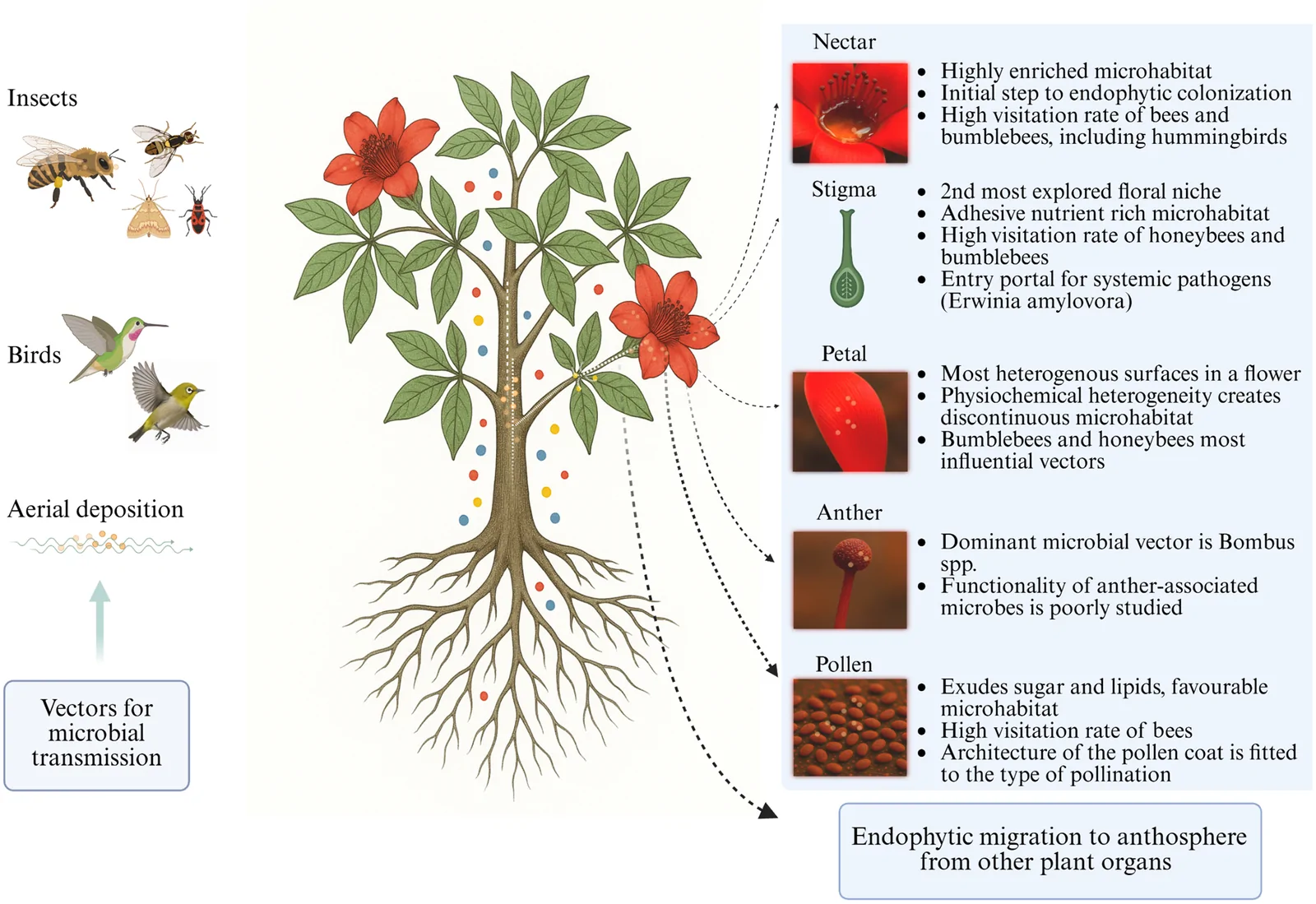
Conceptual diagram describing pathways and major vectors of microbial transmission and colonization in floral organs.

As such, flowers are infested and colonized by different microbial taxa, including both beneficial and pathogenic microbes. There are several documented cases where flowers may serve as a point of entry for pathogenic microbes, such as strawberry (9), pomaceous plants (10), apple (11), blueberry (12, 13), and kiwifruit (14). These pathogenic microbes enter the internal plant tissues via a nectary (15) or stigma (16). Among the most studied ones is *Erwinia amylovora* (17), a causative agent of fire blight disease in apple. It is spread among trees by flower bees, primarily pollinator bees, wind, and rain. When the climate and physiological conditions are favorable and plants are susceptible to the disease, the disease may then spread downward through one diseased flower to the rootstock and kill the tree in one season (17, 18). The previous studies though cleared that flowers are not the final point of microbes but portals. Microorganisms deposited on stigmas, nectaries, or on petal surfaces can invade and colonize plant tissues, and enter the vascular system, intercellular spaces, and contribute to the internal, structured microbial communities of plants (19). Besides external transmission pathways, internal plant-related endophytes may also contribute to the floral microbial communities through systemic movement to reproductive tissues and seeds (20). Flowers serve as the ecological bridge for the transmission of microbes from other plants to internal plant tissues (Fig. 1).

Floral microbes are very important in ecological functions, and it is necessary to know how they get introduced and become established in flowers. Flower microbial assemblages are created through several dispersal mechanisms, which include the following. (i) Insect-mediated dispersion: pollinators, including bees, butterflies, flies, and beetles, infiltrate microbes during their visits; nectar robbers, like carpenter bees (*Xylocopa* spp.), short-tongued bumblebees piercing the corollas, wasps, and ants that do not have to touch the reproductive structures, also play a role in microbial dispersal to the floral tissue. (ii) Airborne deposition: microbial propagules (e.g., spores, cells of bacteria) intervene to land on flowers by wind, rain, or aerosol. (iii) Avian transmission: in this case, microbes are inoculated directly by nectar-feeding birds such as hummingbirds or sunbirds, on beaks, tongues, or feathers. Insect vectors are emerging as a leading cause of floral microbial community composition. This hegemony is owed to the high number of insect flights, proximity, and specificity of floral insect visits. On a single day, pollinators usually come into close association with hundreds of flowers, having their body surfaces covered with microbes, in direct contact with the stigma, nectaries, and anthers (21). Also, numerous pollinators exhibit floral fidelity—the recurrent visiting of flowers of a similar species—which facilitates the regular introduction and compounding of microbial taxa between conspecific plants (22). In addition, the insect-related microorganisms are frequently metabolically adapted to flourish inside flowering habitats, and thereby they are effective colonizers and functional agents. Conversely, the airborne microbes, always present, seem to serve only to initiate background colonization or at an early stage in open flowers exposed to wind or rain (3). These airborne microbes are usually generalists and are less enduring; experimental studies indicate that they are not as impactful regarding the configuration of floral chemistry or pollinator behavior (2). In the meantime, the transfer of microbes with the help of birds is narrower regionally and system-specific. It is in these ecosystems with bird pollination predominating (i.e., tropical forests or alpine habitats) that nectarivorous birds have the potential to be microbial vectors, yet because the frequency of their visits is less and their distribution narrower globally, they are less influential than their insect counterparts (23).

Collectively, these observations indicate that flowers should be considered microbial hubs, with several sources coming together and making point sources of dispersal to microbes both to pollinators and to surrounding plants, and to systemic tissues. From an eco-evolutionary perspective, floral microbiota is structured by interactions among plant traits, pollinator behavior, and environmental filtering. Floral characteristics, such as nectar chemistry, morphology, phenology, and pollination syndrome, act as ecological filters determining which microbial taxa colonize and persist. In turn, microbial modification of floral rewards, scent profiles, and reproductive tissues can influence pollinator behavior and plant reproductive success, potentially generating feedback loops that contribute to trait selection over evolutionary timescales. This review is an attempt to summarize the existing data on the sources and processes of microbial transfer to flowers by comparing the relative importance of insects, birds, and aerial deposition. We especially highlight the importance of insect visitors as the main vectors of microbes and test how the colonization of flora can contribute to systemic microbial transfer in plants. The review seeks to offer an ecological and evolutionary context of floral microbiomes by incorporating insights into the viewpoints of plant ecology, microbiology, and pollination biology.

## STUDY INCLUSION CRITERIA AND REVIEW PROCESS

### Search strategy

To achieve a synthesis of the available empirical research on floral microbiomes, transmission vectors, and the distribution of microbial colonization across the floral organs, we used a systematic literature search, focusing on articles from the following four databases: Web of Science Core Collection, Scopus, PubMed, and Google Scholar, of all publications up to December 2025. We then used a set of Boolean keyword combinations, including those that retrieved both floral tissues and microbial assemblages, such as “floral OR flower OR nectar OR stigma OR pollen OR petal OR anther” AND “microbiome OR microbe* OR yeast OR fungi OR bacteria OR endophyte,” and terms related to transmission, including “pollinator OR bee OR bumblebee OR insect OR bird OR hummingbird OR sunbird OR wind OR airborne.”

### Inclusion

We included studies that met the following criteria: (i) scientific articles; (ii) thesis studies; (iii) research articles but not review articles; (iv) articles published up to December 2025; (v) *in vivo* or *in vitro* studies; (vi) studies which were conducted solely to study the role of targeted vectors (insects, birds, and by air) in microbial transmission to flowers.

### Screening and selection criteria

The titles and abstracts were then screened by the reviewers to allow them to select papers that met the inclusion criteria. The research papers that did not fit the inclusion criteria were not subjected to any further analysis. Articles written in English and containing adequate methodological descriptions to derive information on microbial taxa, sampling, and related vectors were the only eligible ones. Following the screening and selection process, a total of 41 research articles were retained and included in the final synthesis and analysis.

### Data extraction and analysis

We also systematically retrieved information on each study meeting the inclusion criteria on the types of floral parts under investigation (nectar, stigma, petal, pollen, and anther), the type of microbial taxa involved in the study (bacteria, yeasts, and fungi), the primary route of transmission involved in their study (insect vectors, birds, or air deposition), and the specific plant species used in the study and their floral syndromes. To generate the proportional summaries, we generated a heatmap to present the studies on the microbial distribution across floral parts and a bipartite network model to show the interaction of floral parts and vectors on the basis of previous literature. The visualization of networks was done by ggraph with bipartite layouts using R. Data were compiled from published studies reporting bacterial and fungal taxa associated with floral organs, along with corresponding plant species, floral traits, and pollinator information. Microbial and trait data were standardized and expanded into long format, and pollination syndromes were assigned using a rule-based classification integrating floral traits such as corolla depth, flower color, nectar characteristics, and documented floral visitors (24). Microbe-pollination syndrome co-occurrence frequencies were calculated at the study level and visualized as a heatmap to compare microbial associations across pollination strategies. All analyses were conducted in R using tidyverse, igraph, ggraph, and ggplot2 packages. The differences in the distribution of studies across the floral organs were tested by a chi-square goodness-of-fit test, and effect size was determined by Cramér’s V. Microbial deposition patterns, dispersal routes, and colonization possibilities were computed to create conceptual illustrations of the interaction between insects, birds, and air to form floral microbiomes.

## MICROBIAL TRANSMISSION TO FLOWERS

**Fig 2:**
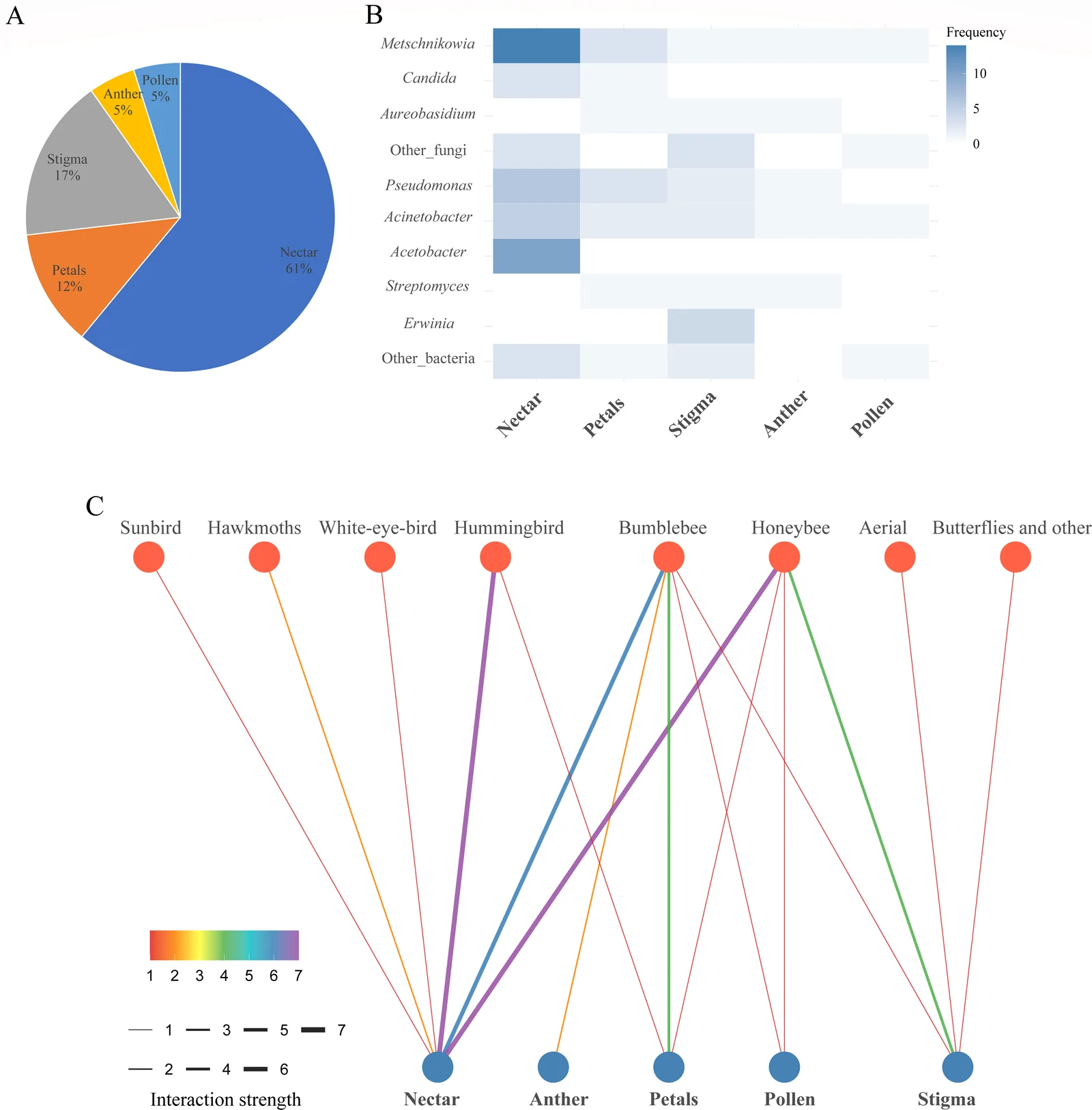
Analysis of floral microbial studies included in this review (*n* = 41). (**A**) Proportion of published studies investigating microbial communities across different floral organs. The distribution differed significantly from an equal expectation (χ² = 45.22, df = 4, *P* < 0.001), indicating research bias toward nectar and stigma-associated microbiota. (**B**) Frequency of major microbial groups across different floral organs. (**C**) Bipartite network showing associations between floral organs and vectors of microbial transmission. Interaction strength reflects the number of studies reporting each interaction.

The bipartite network (Fig. 2A) revealed strong differentiation in how floral parts interact with major transmission vectors, demonstrating that floral microbial study is highly uneven across floral parts.

### Transmission by insects

Insects play a key role in symbiotic relationships between plants and microorganisms. Insects offer a dispersal service to microorganisms between plants and, in exchange, benefit from nutrients provided by the plants. In turn, they pick up microorganisms that can be harmful or beneficial to the plant. Microorganisms can fluctuate floral traits, thereby contributing to the attraction or repellence of a pollinator (25, 26). As shown in previous studies, functionally differentiated fungal communities inhabiting stigmatic exudates and petal nectar mediate plant-pollinator mutualisms in *Monoon laui*, highlighting the importance of organ-specific microbial functions in floral ecology (27). The plant provides resources to the microorganisms, which are transferred by pollinators through ingestion or even attachment. Plants use nutrients in producing resources of nectar and pollen, and more importantly, they are assisted by the pollinators in reproduction. This relationship benefits plants by aiding pollination, while the microorganisms can positively or negatively affect the plants (28).

The bipartite network showed that insects are the most intensively studied vectors connecting to almost all floral organs. Insects, especially honeybees and bumblebees, are central to the microbial exchange in flowers and are extensively studied vectors (Fig. 2C). Bee visitation facilitates the direct inoculation of microbes into floral organs, especially in the case of insects (29, 30). Insect pollinators, especially honeybees and bumblebees, are well recognized as key agents of pollination and are among the most frequently reported vectors of microbial transfer to flowers in the compiled literature (Fig. 2C). Comparative assessments show that bumblebees and honeybees are the most commonly studied microbial vectors, far exceeding other insect visitors such as beetles, as well as bird pollinators such as hummingbirds. These include, in particular, the family Apidae, particularly the honeybee, *Apis mellifera,* because it is a generalist pollinator and a eusocial animal (31). Diet, pattern of floral visitation, and exposure to the environment significantly influence the composition of the bee microbiota. The gut microbiome is one of the best-studied insect-associated microbial communities in honeybees and consists of a relatively stable and core population dominated by Proteobacteria, Firmicutes, and Actinobacteria (32). These microbes perform essential functions in nutrient metabolism, immune response, as well as pathogen resistance, and plenty are also discovered in the floral habitats visited by bees, implying that there may be bidirectional exchange between bees and the flowers (29).

In addition to the presence of typical communities of gut-associated microbes, there are insect pollinators that actively deposit microbial communities on tissues of plants during visitation through their open exoskeleton. The use of pollinator exoskeletons and body hairs, which have long been known as vectors of pollen transport (33, 34), is also an important source of microbial transmission to flowers. The insects are heavily colonized by microbial communities on their exposed exoskeletons; as Ushio et al. (30) estimated that individual insects carry on average 1.22 × 10^6^ microbial cells, with larger body size, bumblebees potentially carrying larger amounts of microbial cells owing to its greater surface area and contact with the floral (35). Allogrooming and food sharing are social behaviors that aid honeybees in microbe distribution throughout colonies and enhance the amount of microbes that can be deposited during floral visits (36, 37). Bees also bring certain taxa during nectar and pollen foraging, e.g., nectar-specialist yeasts like *Metschnikowia reukaufii* and bacteria like *Acinetobacter nectaris* onto floral tissues. Once deposited, such microbes rapidly colonize nectar, alter its chemical composition, decrease the pH through the formation of organic acid, and modify volatile compounds. These alterations influence the microbial community assembly and have also impacted pollinators’ behavior. Previous studies also show that honeybees’ visitation strongly alters the composition of bacterial communities of nectar, indicating that they can be described as active contributors to microbial assembly (38). Transmission through insects also plays a role in the transmission of pathogens. An example is the *Erwinia amylovora*, the causal agent of fire blight, which can be transmitted to flowers by pollinating bees. After deposition on the stigma, bacterium rapidly proliferates, enters the floral cup, and subsequently invades the vascular tissues through the style (39). Insect morphology, behavior, and their related microbiota influence the exchange of microbes, as well as the functional assembly of the floral microbial communities.

Although the bee microbiomes have gained significant focus, knowledge on the non-bee insect vectors (flies, wasps, beetles, moths, butterflies, etc.) remains limited. Other than bees, tiny and ubiquitous pollinators like thrips are capable of scattering bacteria to the flowers, and culturable bacteria have been demonstrated to be more common than fungi in nectar (40). Flies, which have a very different physiology, foraging behaviors, and energetic needs, exhibit more variable microbial associations that reflect environmental acquisition rather than behavioral patterns (41, 42). Recent studies of the hoverfly (*Eristalis tenax*) have identified gut communities enriched in *Orbaceae* and *Lactobacillaceae,* some of which are shared with bee-related microbes but also probably have different ecological roles (42).

There is still limited information on the role of other insects’ pollinators in microbial transfer to the floral organs, such as drone flies, bee flies, beetles, and lepidopterans (butterflies and moths) (28). More studies are needed to explore the microbial acquisition and transmission patterns in these species. Concluding briefly, insect vectors, particularly bees, serve as microbial carriers and agents, influencing the floral microbial composition by being both direct carriers and agents of microbe introduction through behavioral characteristics. Variability of microbial acquisition among social and solitary species, functional diversity of related taxa, and underscores the importance of insect visitation in shaping floral microbiomes. This helps explain why insects are more significant than aerial and bird vectors in most ecosystems for floral microbiota.

### Bird-mediated transmission

Although being less often studied than insect vectors, nectar-feeding birds represent a biologically plausible and ecologically relevant pathway for microbial transmission to flowers. In the Americas, hummingbirds (Trochilidae), and in the Old World, sunbirds (Nectariniidae), represent a biologically feasible and ecologically relevant mechanism of microbial flow to flowers through repeated contact with their beaks, tongues, and feathers, which provides the opportunity for microbial deposition and dispersal between individuals and between species (23). In comparison to insects, birds tend to visit flowers less often and, in most cases, are less selective in their foraging ranges, particularly in a temperate ecosystem. Nevertheless, birds can leave high microbial loads when present because of their relatively large body size, extended time in feeding, and penetration deep into the flowers (2, 43). Bird-pollinated (ornithophilous) flowers are typically characterized by tubular corollas, high nectar volumes, and red or orange coloration traits that can reduce insect visitation and elevate birds as the dominant floral visitors and microbial vectors in these systems (44, 45).

In ecosystems where bird pollination predominates, such as Neotropical cloud forests, alpine regions, tropical islands, and fynbos ecosystems, avian transmission may play a disproportionately important role in shaping floral microbiomes. In these habitats, pollination networks are often simplified and bird-dominated, increasing the ecological influence of avian microbial vectors (46). Empirical studies have reported distinct microbial assemblages in bird-pollinated flowers, suggesting that avian visitation can structure nectar and floral surface microbiomes in ways that differ from insect-pollinated systems (47, 48).

Nectar-feeding birds in ornithophilous systems may be important vectors of microbial transmission in flower visitation, since these systems are provided with high volumes of nectar adjusted to the feeding physiology of birds (49). Hummingbirds have been observed to transfer nectar-related acetic acid bacteria like *Gluconobacter* and *Acetobacter,* which have evolved to live in nectar-high-sugar habitats and have the ability to alter nectar chemistry through sugar metabolism and organic acid synthesis (44, 50). In addition to direct deposition, microbial assembly of bird-associated systems could also be preconditioned by environmental filtering through the diet. For example, nectar in the *Nicotiana glauca* system with *Cinnyris osea* (sunbird) includes toxic pyridine alkaloids (anabasine and nicotine) that select bacteria with the ability to degrade alkaloids. Experimental dysbiosis lowered the efficiency of detoxification, and *in vitro* tests revealed that *Methylorubrum populi*, *Lactococcus lactis*, *Enterobacter hormaechei*, *Chryseobacterium gleum*, and *Kocuria palustris* were active degraders of these compounds (51). These results indicate that microbes can be pre-selected by host diet and nectar chemistry in transmission by birds and thus help assemble and structurally organize floral microbiota. Even though the number of studies dedicated to bee-mediated systems is higher than to bird visitation, the evidence that is available suggests that bird visitation can have a meaningful impact on nectar microbial communities.

Experimental evidence that microbial transfer through birds to flowers occurs is not very decisive but is accumulating. An example is an article by Theron-De Bruin et al. (52), which studied that birds indirectly facilitate the dispersal of fungal spores in *Protea* species flowers through mite vectors, showing multi-partner transmission pathways. Similarly, in an additional study conducted in an urban environment (53, 53), it was found that frequent bird visitation is associated with diverse microbial colonization patterns on flowers, implicating birds as potential microbial contaminants or vectors. Despite these findings, causal links between avian visitation and microbial establishment remain poorly resolved.

**Fig 3:**
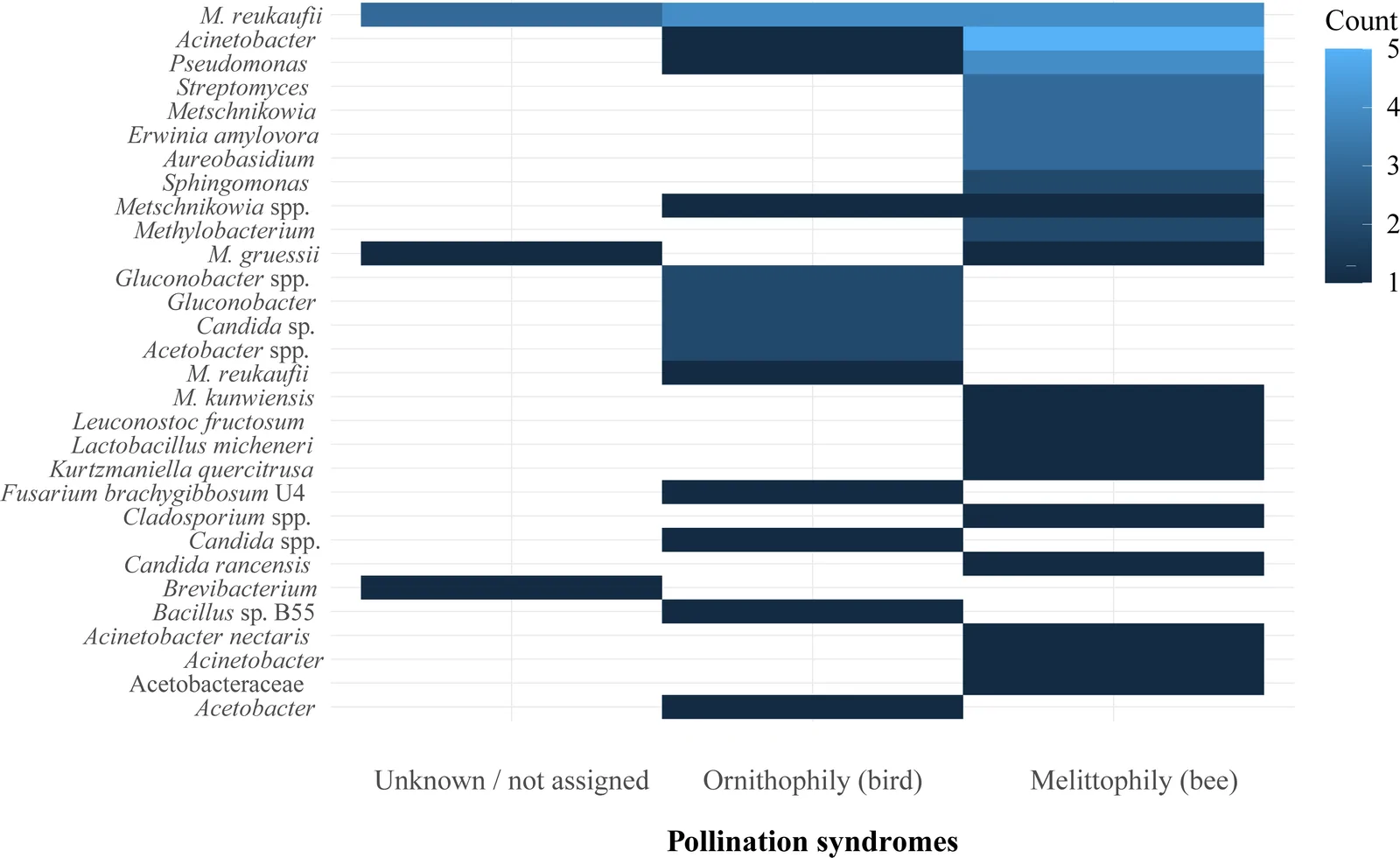
Heat map illustrating the dominant floral microbial taxa associated with pollination syndromes. Tile intensity represents the number of independent study records in which a given microbial taxon was reported within a particular pollination syndrome. Pollination syndromes were assigned using a rule-based classification integrating floral trait descriptors and documented floral visitors.

In conclusion, birds are likely secondary microbial vectors in most temperate plant–pollinator networks; they can act as primary contributors to floral microbiome assembly in ornithophilous systems where insect visitation is limited, similar to our findings (Fig. 3). Their role is therefore context-dependent, highlighting the need for targeted experimental studies in bird-pollinated ecosystems.

### Aerial deposition

Along with the insect- and bird-mediated pathways of transmission, aerial deposition has been described as a significant but comparatively underestimated vector for microbial colonization of floral tissue. Microbial propagules such as bacteria, fungal spores, and yeast cells are dispersed by the wind in the form of wind currents, dust, aerosols, and rain splash (2). These propagules can settle on exposed floral surfaces, such as stigmas, petals, anthers, and nectar reservoirs, particularly in flowers with open or shallow morphologies.

The only available study investigating airborne transmission reported a stronger association with stigma than with other floral organs, possibly reflecting the greater exposure of stigma to passive microbial deposition. However, additional studies are needed to confirm this pattern (Fig. 2C). The propagules can also be deposited on the floral surface, i.e., petals, stamens, or nectar reservoirs, especially in open or exposed floral morphologies. Despite the common perception that it is a passive process, the effect of aerial deposition can substantially influence early microbial colonization, especially in unopened flowers or in flowers experiencing limited or no animal visitation (54). The abundance and composition of the airborne microbial communities depend largely on the wind speed, humidity, rainfall, seasonality, and altitude. The airborne microbial load is usually high in open-canopy habitats, dry climatic conditions, and cities or agricultural settings than in the dense forest systems (55).

Notably, as compared to a vector-based approach, deposition of aerial contaminants is not host- or species-specific. As a result, it primarily introduces generalist or environmentally resistant taxa that are subsequently filtered by the physicochemical properties of floral tissues, such as nectar chemistry or surface metabolites (2, 7). Airborne microbial communities are often dominated by bacterial phyla, i.e., Actinobacteria, Proteobacteria, and Firmicutes, and fungal spores belonging to Ascomycota and Basidiomycota. Nevertheless, it is these higher taxonomic groups that contain taxa that are implicated in pollinators; distinctions between airborne and pollinator-mediated communities tend to arise more at finer taxonomic levels, relative abundance, and persistence within floral habitats. Many of these microbes might stay in dormant or transient states, and some might be able to colonize floral tissues under conditions of favorable environment or where there is low competition (56).

Deposition, however, is not the sole determinant of community structure. The high sugar concentration, osmotic stress, UV light, and antimicrobial secondary metabolites are components of nectar chemistry that serve as a powerful environmental filter to selectively allow persistence of stress-tolerant and metabolically flexible taxa (57). As an example, it has recently been shown that even though there are numerous sources of environmental microbes, the floral communities are not randomly built by the confluence of dispersal and filtering by nectar (58). Taken together, these results suggest aerial deposition as an initial inoculum; however, deterministic selection in the microenvironment of the flower can ultimately control the structure of microbial communities.

Experimental studies support the view that airborne deposition provides a baseline inoculum rather than a dominant driver of floral microbiome assembly. Several pollinator-exclusion experiments have shown that flowers isolated from animal visitors harbor lower microbial abundance and diversity than exposed flowers (7, 47). For example, Fridman et al. (3) demonstrated that caged flowers accumulate microbial communities largely derived from airborne sources, but these communities are less dense and compositionally distinct from those in pollinator-visited flowers. Similarly, high-throughput sequencing of air samples above flowering plants has revealed viable microbial taxa that overlap with floral communities, reinforcing the idea that the atmosphere serves as a reservoir rather than a primary shaping force (59).

Therefore, aerial deposition of microbes in the air forms a background, non-pollinator route that colonizes exposed floral tissues. However, its influence is generally secondary to that of insects and birds, which deliver larger, more consistent, and often better-adapted microbial inocula to flowers.

## FLORAL NICHES AND MICROBIAL COLONIZATION

Floral organs represent distinct ecological niches that differ markedly in structure, chemistry, exposure, and interaction with pollinators. As a result, microbial colonization within flowers is strongly organ-specific. The heatmap (Fig. 2B) showed significant variations in the distribution of microbial studies across floral organs, showing that the colonization of the floras by the microbes is strongly organ-specific.

Across the literature, microbial studies are unevenly distributed among floral organs (Fig. 2A), reflecting an equal expectation (χ^2^ = 45.22, df = 4, *P* < 0.001; Cramér’s V = 0.525), indicating substantial research bias toward nectar and stigma microbiota. These disparities likely arise from differences in accessibility, microbial abundance, and perceived ecological relevance, but they also obscure a comprehensive understanding of how microbes colonize entire flowers.

### Nectar

All the collected studies revealed that the most thoroughly researched microhabitat in the flora is nectar (61%) of all reported floral microbiome studies (Fig. 2A). The highest frequency and diversity of microorganisms in nectar was seen with the predominance of the yeast *Metschnikowia* and bacterial groups, i.e., *Acetobacter*/*Gluconobacter*, *Pseudomonas*, and *Acinetobacter* (Fig. 2B). This pattern aligns with previous studies which studied nectar, which is a nutrient-enriched interface of plant, pollinator, and microbial interactions. The majority of angiosperm flowers have nectaries, special glands or glandular areas where they release a sugar-containing nectar, which acts as an incentive to pollinators. These microenvironments are highly humid and rich in carbon substrates and thus ideal for the development of microbes. Nevertheless, physicochemical factors of the nectar (high osmotic pressure, low nitrogen content, and the presence of antimicrobial secondary metabolites) exert high selective pressures to define the existence of microbial taxa (60). Recent studies also emphasize the knowledge that the microorganisms themselves also contribute to nectar chemistry, affecting the composition of sugars and secondary metabolites (61). The largest group of chemical compounds in nectar that have been known to be in strong interaction with microbes are those proteins in nectar, which are referred to as nectarins. It has been suggested that nectarins are capable of inhibiting the growth of microbes in nectar by generating high levels of hydrogen peroxide (62).

Despite these defenses, nectar remains one of the most consistently colonized floral niches. Yeasts, particularly *Metschnikowia reukaufii*, are ubiquitous in nectar across diverse plant taxa, including *Clematis akebioides*, *Aquilegia vulgaris*, *Erysimum mediohispanicum*, and *Helleborus foetidus* (26, 63, 64). Basidiomycetous yeasts are comparatively rare, reflecting differential tolerance to nectar conditions.

Bacterial communities in nectar are similarly shaped by physicochemical constraints, with members of the *Acetobacteraceae* frequently dominating due to their adaptation to high-sugar, low-pH environments (65, 66). Insect visitors, particularly honeybees and bumblebees, represent the most frequently reported vectors introducing these microbes into nectar, as evidenced by the strong association between insect-mediated transmission and nectar colonization in the bipartite network analysis (Fig. 2C). This pattern aligns with previous studies demonstrating that pollinator visitation and local ecological context strongly structure nectar microbial communities (7, 67, 68). Birds can also contribute to nectar microbiota in specific systems, but their overall influence appears more limited and regionally restricted (47, 69).

Beyond serving as a microbial habitat, nectar may function as a gateway for microbial entry into internal plant tissues. Pathogens, such as *Erwinia amylovora*, can enter through nectar or stigma surfaces and subsequently colonize vascular tissues, leading to systemic disease (8, 10). Conversely, beneficial taxa, including *Acinetobacter* and Bacillus species, have been detected in both nectar and internal plant tissues, suggesting that nectar colonization may represent an initial step toward endophytic establishment (70, 71). Collectively, these findings position nectar as both an incubator and a conduit for microbial movement within plants.

### Stigma

The stigma represents a critical interface between reproduction and microbial colonization, acting as a recipient of pollen, pollinator contact, and airborne propagules. It is the second most frequently studied floral niche, accounting for approximately 17% of published records (Fig. 2A). Structural differences between dry and wet stigmas strongly influence microbial colonization. Dry stigmas are characterized by papillae covered by a cuticle and pellicle, whereas wet stigmas secrete lipid- and protein-rich exudates that form adhesive, nutrient-rich microhabitats conducive to microbial growth (72, 73).

Stigma-associated microbial communities commonly include bacterial genera such as *Pseudomonas*, *Acinetobacter*, and *Erwinia,* and yeasts such as *Metschnikowia* and *Aureobasidium* (9, 74). These microbes are predominantly introduced by pollinating insects, particularly honeybees (*Apis mellifera*) and bumblebees (*Bombus* spp.), although airborne deposition contributes a basal inoculum (Fig. 2C). Besides these endogenous influences, external forces of the environment like pollution may also alter microbial communities related to stigma, as was recently shown in the case of *Orobanche lutea* (75, 76).

Importantly, the stigma is a well-established portal for systemic plant infection. Erwinia amylovora, the causative agent of fire blight, colonizes stigmatic surfaces before invading internal tissues, making the stigma a focal point for disease initiation (8, 10). As such, stigmas function both as microbial sinks and as gateways through which epiphytic populations can transition into endophytic or vascular infections.

### Petals

Petals constitute one of the most heterogeneous floral surfaces, varying widely in color, texture, morphology, orientation, and microtopography. These features generate fine-scale gradients in temperature, moisture, ultraviolet exposure, and nutrient availability, which together shape microbial colonization patterns (77). Despite their ecological relevance, petals remain comparatively understudied, accounting for only approximately 12% of floral microbiome studies (Fig. 2A). Pollinators are the major drivers of microbiomes in petals, and these involve the transfer of bacteria and fungi through the body surfaces, mouthparts, beaks, legs, and pollen baskets of pollinators. Within this context, bumblebees and honeybees come out as the most influential vectors shaping petal microbiomes across floral systems (Fig. 2C). Evidence has been obtained from several plant systems that petals often acquire microbial taxa in a pollinator-specific manner. For example, in *Exacum affine*, the composition of the petal microbiome is affected by *Bombus impatiens*, which spreads yeasts like *Metschnikowia gruessii* and *Candida rancensis*, as well as lactic acid-producing bacteria like *Leuconostoc fructosum* and *Lactobacillus micheneri* (78). Evidence of pollinator-mediated transfer of microbes on the surface of the petals of *Clematis apiifolia* shows that two bee species, *Bombus diversus* and *A. mellifera*, help transfer *Sphingomonas* and *Methylobacterium* (30). In the case of *Fragaria x ananassa*, insect visitors such as *Bombus impatiens* and *A. mellifera* deposit yeasts (*Metschnikowia*, *Aureobasidium*) and bacteria (*Pseudomonas*, *Acinetobacter*) onto petal surfaces, whereas *Bombus terrestris* has been shown to deposit *Streptomyces* on petals (9). In addition, work done on *Mimulus guttatus* shows that bees and hummingbirds contribute to the establishment of *Pseudomonas* and *Acinetobacter* on petal tissues (22). Petal-associated microbes tend to concentrate in regions of frequent pollinator contact, such as nectar guides and basal petal areas, suggesting a fine-scale spatial coupling between floral morphology and microbial inoculation. These patterns underscore that petals function as active ecological interfaces rather than passive surfaces. However, the functional consequences of petal-associated microbiomes, including their roles in scent modulation, pathogen exclusion, and pollinator learning, remain poorly resolved and present a major opportunity for future research.

### Pollen

Pollen grains provide a unique but underexplored microbial niche, accounting for only approximately 5% of floral microbiome studies (Fig. 2A). Following anther dehiscence, pollen surfaces often exude sugars and lipids that facilitate adhesion to stigmas and promote pollen tube germination, while simultaneously creating microhabitats conducive to microbial colonization (79). For instance, concentrated assemblages of Gammaproteobacteria and clustered populations of Betaproteobacteria have been recorded within the floral environment (5). Microbial communities associated with pollen include both bacteria and fungi (80), with reported taxa such as *Bacillus*, *Penicillium*, *Botrytis*, and members of the Gammaproteobacteria and Betaproteobacteria (5, 81). Pollinators, particularly bees, play a central role in transferring microbes to pollen grains through repeated contact during foraging and pollen collection. For example, in controlled experimental studies using *Diplacus (Mimulus) guttatus*, *Bombus impatiens* has been shown to deposit the yeast *Metschnikowia reukaufii* and the bacterium *Acinetobacter nectaris* onto pollen surfaces (82). The structure of the pollen coat itself may act as an ecological filter shaping microbial colonization. Insect-pollinated species typically produce pollen with thicker, lipid-rich coatings, whereas wind-pollinated species tend to have thinner pollen walls (83). These structural differences likely influence microbial attachment, persistence, and community composition. Given pollen’s central role in plant reproduction and its extensive handling by pollinators, the limited attention to pollen-associated microbiomes represents a significant gap in current floral microbiome research.

### Anthers

Whereas the microflora associated with nectar and stigma have been relatively well studied, anthers, the pollen-bearing structures of the flower, also contain unique assemblages of microbes, and these are much less well studied (corresponding to only 5% of studies in our data set; Fig. 2A). Despite their limited study, anthers host distinct microbial assemblages that may influence pollen viability, pathogen suppression, and microbial exchange among floral organs. Available studies indicate that anther-associated communities commonly include bacterial taxa such as *Streptomyces* and *Pseudomonas*, as well as yeasts like *Metschnikowia* and *Aureobasidium* (9, 74) (Table 1). The deposition and horizontal dispersion of such microorganisms are carried out through the visitation of insect pollinators, especially by bumblebees, which are among the most frequently studied microbial vectors in our study. This trend is probably a result of research attention to these taxa instead of established ecological dominance. The functionality of anther-associated microbes is as yet only poorly defined, although experimental studies exist that suggest that they may regulate pollen viability, have antagonistic effects against phytopathogens, or get involved in the microbial exchange between pollen and floral parts (84–87). Accordingly, this niche represents an interesting research area that needs to be investigated systematically in order to understand its contribution to both floral and systemic microbiomes.

**TABLE 1:** Summary of studies reporting microbial transmission to flowers

Parts	Plant	Fungi	Bacteria	Floral visitors	Floral syndromes	Reference
Nectar	*Clematis akebioides*	*Metschnikowia reukaufii* (yeast)		Bumblebee	Tubular bell-shaped flower, visible color	(26)
Nectar	*Mimulus aurantiacus*	*M. reukaufii* (yeast)		Anna’s hummingbird (*Calypte anna*) and Allen’s hummingbird (*Selasphorus sasin*)	Bright orange/red color, tubular flower shape	(47)
Nectar	*Salvia microphylla*	*M. reukaufii* (yeast)	*Acetobacter*, *Gluconobacter*	Anna’s hummingbird (*Calypte anna*) and Allen’s hummingbird (*Selasphorus sasin*)	Bright red to magenta flowers, bilateral symmetry, and tubular corolla	(65)
Nectar	*Salvia greggi*	*M. reukaufii*; *Candida* sp.	*Acetobacter* spp., *Gluconobacter* spp., minor *Fructobacillus* spp.	Anna’s hummingbird (*Calypte anna*) and Allen’s hummingbird (*Selasphorus sasin*)	Tubular red/pink flowers	(65)
Nectar	*Leonotis leonurus*	*Metschnikowia* spp. and other non-dominant yeasts	*Gluconobacter* spp., *Acetobacter* spp., occasional *Pseudomonas* spp.	Anna’s hummingbird (C*alypte anna*) and Allen’s hummingbird (*Selasphorus sasin*)	Large, bright orange, tubular flowers in whorls; strong nectar flow	(65)
Nectar	*Salvia darcyii*	*M. reukaufii*, *Candida* sp.	*Acinetobacter*, *Gluconobacter*, *Pseudomonas*	Anna’s hummingbird (*Calypte anna*) and Allen’s hummingbird (*Selasphorus sasin*)	Deep red flowers, large tubular corolla	(65)
Nectar	*Kniphofia spp*.	*M. reukaufii*, *Candida* spp.	*Proteobacteria* (esp. *Acetobacter*, *Pseudomonas*, *Sphingomonas*); some *Firmicutes*	Anna’s hummingbird (*Calypte anna*) and Allen’s hummingbird (*Selasphorus sasin*)	Tubular red/orange/yellow flowers	(65)
Nectar	*Nicotiana attenuata*	*Fusarium brachygibbosum U4*	*Bacillus* sp. B55	Hummingbird (primarily Anna’s hummingbird, *Calypte anna*) and hawkmoths	Tubular red-orange flowers	(70)
Nectar	*Asclepias curassavica*	*Metschnikowia* spp.	*Acinetobacter* and *Pseudomonas*	Honeybees, Monarch butterflies, and carpenter bees	Bright red/orange sepals and yellow petals	(68)
Nectar	*Helleborus foetidus*	*M. reukaufii* (yeast)		*Bombus terrestris* and *Apis mellifera* (honeybee)	Greenish-purple flowers, cup-shaped open corolla	(64)
Nectar	*Helleborus foetidus,*	*M. reukaufii* (yeast)		*Bombus terrestris* (buff-tailed bumblebee)	Bell-shaped, green to purplish-green	(63)
Nectar	*Helleborus foetidus*	*M. reukaufii* (yeast)		Bumblebee	Greenish-bell-shaped flowers	(7)
Nectar	*Helleborus foetidus*	*M. reukaufii*, *M. gruessii*		Bumblebee	Greenish-bell-shaped flowers	(88)
Nectar	*Malus x domestica*		Undibacterium and Brevibacterium	Honeybees and other visiting insects	White to pink petals, actinomorphic	(66)
Nectar	*Pyrus communis*				Typically white, actinomorphic (radial symmetry)	(66)
Nectar	*Persea americana*		*Pseudomonas* and *Acinetobacter*	*Apis mellifera* (honeybees)	Greenish-yellow, small (5–10 mm across), inconspicuous, paniculate	(71)
Nectar	*Eurya japonica*	Mixed yeast communities		*Zosterops japonicus* (Japanese white-eye)	White to pale-pink bowl-shaped corolla 8–10mm	(69)
Nectar	*Leptospermum scoparium*	*M. reukaufii* and *M. kunwiensis* (yeast)	*Acetobacteraceae*	*Apis mellifera* (honeybees) and native solitary bees	Bowl-shaped, white/pink petals	(61)
Nectar	*Amygdalus communis*		*Methylobacterium rhodesianum* and *Methylobacterium populi*	*Apis mellifera* (honeybee)	Cup- or saucer-shaped, pink or white, five-petaled	(38)
Nectar	*Citrus paradisi*		*Erwinia amylovora* and *Klebsiella oxytoca*	*Apis mellifera* (honeybee)	Star-shaped, white, and fragrant, five-petaled	(38)
Nectar	*Datura wrightii*	*Pseudozyma*, *Candida, Cryptococcus*	*Rosenbergiella*, *Pantoea, Enterobacter*	Hawkmoths	White tubular, night-blooming, strong scent	(89)
Nectar	*Agave palmeri*	*Aureobasidium*, *Thyronectria*, *Alternaria*	*Rosenbergiella*, *Pantoea, Enterobacter*	Hawkmoths and nectar-feeding bats	Large floral display, copious nectar production, night-blooming	(89)
Nectar	*Nicotiana glauca*		*Methylorubrum populi*, *Lactococcus lactis*	*Cinnyris osea* (sunbird)	Long tubular corolla, stamens shorter than stigma, bird-pollinated	(51)
Nectar	*Pulmonaria officinalis* L.		*Actinobacteria*, *Firmicutes*, *Proteobacteria*	*Bombus terrestris*, *Bombus pratorum*	Tube-shaped corolla, early spring flowering, nectar at bottom of tube	(67)
Nectar	Co-flowering species	*Metschnikowia*, *Candida*	*Acinetobacter*, *Rosenbergiella*, *Pseudomonas*	Thrips (*Frankliniella occidentalis*)	Species with long corollas, insect-pollinated systems, species exposed with nectar	(40)
Petals	*Exacum affine*	*Metschnikowia gruessii* and *Candida rancensis*	*Leuconostoc fructosum* and *Lactobacillus micheneri*	*Bombus impatiens* (bumblebee)	Violet to purple, symmetrical	(78)
Petals	*Clematis apiifolia*		*Sphingomonas* and *Methylobacterium*	*Bombus diversus* and *Apis mellifera*	Pure white petals, loose raceme	(30)
Petals	*Fragaria × ananassa*	*Metschnikowia* and *Aureobasidium*	*Pseudomonas* and *Acinetobacter*	*Bombus impatiens* (eastern bumblebee) and *Apis mellifera*	Pure white petals, radially symmetric flowers	(74)
Petals	*Fragaria × ananassa*		*Streptomyces*	*Bombus terrestris* (buff-tailed bumblebee)	Bright white petals and radially symmetric flowers	(9)
Petals	*Mimulus guttatus*		*Pseudomonas* spp. and *Acinetobacter*	*Bombus vosnesenskii* and hummingbirds (Anna’s hummingbird, *Calypte anna*)	Bright yellow corolla, tubular hermaphroditic flowers	(22)
Stigma	*Malus domestica*		*Erwinia amylovora*	*Apis mellifera* (honeybees)	Umbel-like corymb of 5–6 white, saucer-shaped flowers	(8)
Stigma	*Fragaria × ananassa*		*Streptomyces*	*Bombus terrestris* (buff-tailed bumblebee)	Bright white petals, radially symmetric flowers	(9)
Stigma	*Malus domestica*		*Erwinia amylovora*	Insects (especially honeybees)	Pure-white petals that strongly reflect UV light	(16)
Stigma	*Fragaria × ananassa*	*Metschnikowia* and *Aureobasidium*	*Pseudomonas* and *Acinetobacter*	*Bombus impatiens* (eastern bumblebee) and *Apis mellifera*	Pure white petals, radially symmetric flowers	(74)
Stigma	*Monoon laui*	*Kurtzmaniella quercitrusa* and *Cladosporium* spp.		Nitidulidae (sap beetles) and Drosophilidae (fruit flies)	Thick, creamy-yellow petals, trimerous flowers 25–30 mm in diameter	(27)
Stigma	*Orobanche lutea*	*Rhodotorula* (yeast)	*Methylobacterium*, *Sphingomonas*	Airborne and traffic pollution	Vivid yellow corolla with darker yellow venation, tubular flowers	(76)
Stigma	*Malus domestica*		*Erwinia amylovora*	*Apis mellifera* (honeybee)	Pure white petals, radially symmetric	(10)
Anther	*Fragaria × ananassa*		*Streptomyces*	*Bombus terrestris* (buff-tailed bumblebee)	Bright white petals, radially symmetric flowers	(9)
Anther	*Fragaria × ananassa*	*Metschnikowia* and *Aureobasidium*	*Pseudomonas* and *Acinetobacter*	*Bombus impatiens* and *Apis mellifera*	Pure white petals, radially symmetric flowers	(74)
Pollen	*Mimulus guttatus*	*M. reukaufii* (yeast)	*Acinetobacter nectaris*	*Bombus impatiens*	Magenta-pink corolla with prominent yellow, tubular flowers	(82)
Pollen	*Forage plants*		*Spiroplasma*	*Apis mellifera* (honeybee)	Open corollas, abundant pollen, and accessible anthers	(80)

## FLOWER ATTRACTIVENESS AND PHENOTYPE CHANGE DUE TO MICROBES

Most of the studies on floral microbiomes have been on nectar, then stigmas, petals, anthers, and pollen, as a result of research bias on the easy and accessible floral niche of nectar (Fig. 2A). This distribution highlights the historical emphasis of the study of the microbes of the flora on nutrient-enriched surfaces, of which the microbial colonization is most pronounced, which forms the basis of subsequent investigation of their impacts on pollinator behavior and plant fitness. In this section, we synthesize evidence showing how microbes modify floral traits and how these effects vary across pollination syndromes, with particular emphasis on bee- and bird-pollinated systems (Fig. 3).

### Impact of microbes on floral rewards of pollinators

Food rewards are generated by most plants that are attractive to pollinators, and the quality and quantity of rewards can be changed both directly and indirectly by microbes. Microorganisms inhabiting floral tissues can modify both the quantity and quality of these rewards through metabolic activity, thereby influencing pollinator preference and plant fitness (2). Our synthesis reveals that bee-pollinated (melittophilous) flowers harbor a broader and more frequently reported assemblage of nectar-associated bacteria and yeasts than bird-pollinated (ornithophilous) flowers (Fig. 3). Dominant microbial taxa in melittophilous systems include bacterial genera, such as *Acinetobacter*, *Pseudomonas*, *Sphingomonas*, and *Methylobacterium*, as well as nectar-specialist yeasts, particularly *Metschnikowia* and *Candida*. These microbes are well adapted to metabolize nectar sugars and are repeatedly introduced by frequent bee visitation, reinforcing their persistence and functional impact (5, 7).

Microorganisms living on nectar directly change chemical characteristics of nectar by means of their metabolic activity. In addition, microbial metabolism can modify other nectar constituents, including amino acids and organic acids (63). In addition, the concentration and composition of amino acids, organic acids, and other chemical makeup are also dependent on microbial metabolism, which can vary based on the identity of the microbe (5, 50). Such changes are particularly relevant in melittophilous flowers, where frequent visitation by bees facilitates repeated microbial inoculation and dispersal, reinforcing microbial persistence and metabolic impact.

In contrast, ornithophilous flowers tend to host a narrower subset of nectar-adapted microbes, including acetic acid bacteria, such as *Gluconobacter* and *Acetobacter*, and yeasts like *Metschnikowia* spp.; however, these taxa are not exclusive to bird-pollinated systems and are also widely studied in bee-pollinated flowers (Fig. 3). These taxa are well adapted to high-volume, dilute nectar environments typical of bird-pollinated flowers and may reflect lower visitation frequency but greater nectar turnover per visit. Experimental work supports this pattern, showing that nectar colonization by acetic acid bacteria can reduce nectar volume and accelerate floral senescence, thereby indirectly influencing pollinator foraging dynamics (50).

Microbial effects on pollen rewards appear more context-dependent. While most non-pathogenic microbes colonize pollen surfaces after anther dehiscence and are unlikely to affect pollen production directly, they may influence pollen quality or availability when pollen is exposed to nectar or microbial metabolism. In rare cases, such as infection by *Microbotryum violaceum*, pathogens can entirely replace pollen with fungal spores, resulting in complete host sterilization (90). However, such dramatic outcomes represent exceptions rather than the dominant pattern observed in pollinator-mediated microbial assemblages.

### Microbes regulate floral display and foraging

Pollinator foraging is frequently based on floral display characteristics (e.g., floral scent, color, and morphology) rather than direct evaluation of rewards (77, 78). Floral microbes can modify these traits by producing volatile organic compounds or by metabolizing plant-derived floral volatiles, thereby altering the chemical signals perceived by pollinators (25, 91). Both bacteria and fungi inhabiting nectar or petal surfaces can contribute novel compounds to floral scent profiles or degrade existing floral volatiles, leading to changes in pollinator attraction or avoidance (2).

Furthermore, apart from its modification using volatile blends, microorganisms inhabiting nectar can, via their metabolism, modify its chemical composition. Yeasts and bacteria, which are found in nectar, can alter its chemical composition and lower the pH by producing organic acids, which in turn affects both the composition and physicochemical properties of the nectar (5, 50, 92). These changes can either attract or drive away pollinators based on the identity of the microbes; honeybees, in the example, avoid using nectar that has been colonized by some bacterial species and use nectar that has been colonized by yeast (93).

Microbial taxa frequently associated with bee-pollinated flowers, including *Metschnikowia reukaufii* and several bacterial genera, are known producers of esters, alcohols, and other volatile compounds that overlap with plant-derived floral scents. For example, nectar yeasts can produce 2-phenylethanol, a compound that attracts bumblebees at low concentrations but exhibits antimicrobial activity at higher levels (94). Similarly, bacterial volatiles, such as acetoin, have been linked to altered pollinator visitation, although their direct role as attractants remains under investigation (94).

The predominance of these microbes in melittophilous systems (Fig. 3) suggests that microbial modification of floral scent is particularly pronounced in flowers experiencing frequent insect visitation. In contrast, bird-pollinated flowers rely more heavily on visual cues and nectar volume than on scent, potentially reducing the relative importance of microbial contributions to volatile blends. Nevertheless, nectar-associated microbes in ornithophilous systems may still influence floral attractiveness indirectly by affecting nectar persistence, renewal rates, and floral longevity (44, 47, 50). The variation in the composition of nectar microbes among the systems of pollination and among different plant species, including night-blooming desert plants, also confirms the context-specific structuring of the floral microbiota (89).

### Ecological consequences

Microorganisms are suggested to have an important role in the large variation observed in floral traits and plant-pollinator interactions. Previous studies have shown that microbial abundance and composition can explain a large proportion of variation in nectar sugar concentration and composition among plant species, accounting for up to 65% of observed variance in some systems (7). Recent syntheses also emphasize the idea that nectar-related microbial diversity has the potential to impact pollination dynamics, and it can be a contributor to the processes of pathogen suppression and biocontrol, which means that they have an even more significant ecological impact (95). In our compiled data set, patterns in the frequency of microbes are different between pollination syndromes (Fig. 3). Bee-pollinated flowers tend to be more associated with diverse nectar microbes than bird-pollinated systems do. However, these observations are based on the frequencies that are reported and show the need for further quantitative analyses to test effects at the level of syndromes.

By modifying floral phenotype, microbes can act as indirect signals of reward quality or quantity to pollinators. Pollinators such as honeybees, bumblebees, hummingbirds, and other insect visitors may exhibit attraction to or avoidance of microbially altered nectar depending on microbial identity, concentration, and metabolic products (23). While some microbial colonization reduces pollinator visitation, other microbes enhance attractiveness. For instance, nectar-specialist yeasts of the genus *Metschnikowia* have been shown to increase visitation rates by bumblebees and hoverflies in multiple systems (26, 88, 96).

Inoculation experiments demonstrate that *Metschnikowia reukaufii* can increase pollinator visitation in crops such as pear (*Pyrus communis*) and wild species such as *Clematis akebioides*, although these increases do not always translate into higher fruit or seed set (96). These findings align with the strong association between *Metschnikowia* species and melittophilous flowers observed in our study (Fig. 3) (Table 1).

Overall, evidence demonstrates that microbes in flowers alter flower characteristics and affect plants and their relationship with pollinators instead of merely fostering their presence. Pollination syndromes act as ecological filters shaping microbial assemblages, which in turn influence floral traits, pollinator behavior, and in some cases, plant reproductive fitness. Experimental studies have found that nectar microbes can alter fruit set and seed production, and the extent of the change depends on the identity of the microbe and the environment surrounding the plant (96). Integrating microbial ecology into pollination biology thus reveals microbes as dynamic contributors to the extended floral phenotype rather than passive inhabitants.

## CONCLUSION

This review highlights flowers as dynamic microbial hubs where plant traits, pollinator behavior, and microbial communities intersect. Floral microbial assembly is strongly organ-specific, with nectar and stigmas serving as primary sites of microbial accumulation and transmission. Insect pollinators, particularly bees and bumblebees, emerge as the dominant vectors of microbial dispersal, linking multiple floral organs through repeated and targeted visitation. Bird-mediated transmission, while geographically restricted, plays a significant role in ornithophilous systems, whereas airborne deposition contributes a persistent but largely non-specific background inoculum. Across macroevolutionary timescales, repeated adaptations in floral traits and pollination strategies have led to the reorganization of the microbial transmission networks on a significant scale. The evolution of specialized floral morphologies, such as poricidal anthers, alters the presentation of pollen, nectar accessibility to the visitor, and visitor filtering, thereby limiting or expanding the potential microbial vectors. Similarly, insect foraging behavior and specialization influence patterns of microbial dispersal among plant species, further contributing to microbial diversification. Together, these pathways of microbial transfer and evolutionary processes suggest that contemporary floral microbiomes are shaped not only by ecological interactions but also by the evolutionary history of traits that structure plant–pollinator–microbe interactions over deep temporal scales.

Despite recent advances, several critical research gaps remain. First, there is a pronounced bias toward nectar-focused studies, leaving pollen-, anther-, and petal-associated microbiomes comparatively underexplored. Second, most empirical work studies have focused on bee-mediated transmission, resulting in a limited understanding of the microbial roles of non-bee insects, birds, and mixed pollination systems. Third, although evidence suggests that flowers can serve as entry points for microbial entry into internal plant tissues, the mechanisms, frequency, and functional consequences of systemic microbial movement remain poorly understood. Additionally, few studies have integrated microbial function with plant fitness outcomes, pollinator health, or long-term ecological dynamics.

Future research should move beyond descriptive surveys to experimentally test how specific microbial taxa influence floral phenotypes, pathogen dynamics, and plant fitness. For example, controlled inoculation experiments could investigate whether *Metschnikowia reukaufii* differentially alters nectar pH, sugar composition, and volatile production in bee- versus bird-pollinated plant species, and whether these changes subsequently affect pollinator visitation rates and reproductive outcomes. Similarly, manipulative field studies could evaluate whether bee-mediated microbial deposition, particularly by commonly studied taxa such as honeybees and bumblebees, enhances or suppresses floral pathogens, such as *Erwinia amylovora*, under natural conditions. Testing these hypotheses across contrasting pollination syndromes and environmental contexts would clarify whether pollinator identity systematically shapes floral microbiomes and their functional consequences for plant health and reproduction. Addressing these questions will advance mechanistic understanding of how floral microbiomes connect pollinators, plants, and ecosystems, with important implications for conservation, crop management, and plant health.
